# Culture‐negative group B streptococcal pericarditis: A case report and literature review of the diagnostic use of polymerase chain reaction

**DOI:** 10.1002/ccr3.2017

**Published:** 2019-02-05

**Authors:** Takahiro Tsushima, Natsuko Ishii, Suguru Matsuzaka, Keith Armitage, Kiyofumi Ohkusu, Ivor Cammack, Akira Yamada, Yuichiro Mori, Shunsuke Sasaki, Kentaro Hayashi, Yoshimoto Serizawa

**Affiliations:** ^1^ Department of Medicine University Hospitals Cleveland Medical Center Cleveland Ohio; ^2^ Division of Cardiology Teine Keijinkai Hospital Sapporo Japan; ^3^ Department of General Internal Medicine Teine Keijinkai Hospital Sapporo Japan; ^4^ Division of Infectious Diseases and HIV Medicine, Department of Medicine University Hospitals Cleveland Medical Center Cleveland Ohio; ^5^ Department of Microbiology Tokyo Medical University Tokyo Japan; ^6^ Division of Cardiac Surgery Teine Keijinkai Hospital Sapporo Japan

**Keywords:** 16S rRNA, bacterial pericarditis, group B streptococci, pericardiostomy, polymerase chain reaction

## Abstract

Although conventional microbiology cultures may be negative, polymerase chain reaction (PCR) can effectively identify both typical and atypical microorganisms. With careful interpretation, PCR could become the gold‐standard diagnostic test for culture‐negative bacterial pericarditis.

## 1 CASE PRESENTATION

Despite medical progress, bacterial pericarditis remains a life‐threatening condition. In this case, despite negative bacterial culture of the pericardial effusion, polymerase chain reaction (PCR) successfully identified the primary microorganism as group B streptococci. We performed a literature search and summarized relevant articles describing the use of PCR in this setting.

An 82‐year‐old woman with a past medical history of coronary artery disease and type 2 diabetes mellitus (HbA1c 7.6%) presented with a 1‐week history of gradually worsening substernal pleuritic chest pain and fever. The pain was exacerbated with coughing, deep inspiration, and leaning forward. There were no other cardiac or respiratory symptoms. She was independent and lived alone. She had been a heavy smoker in the past, but denied any other recreational drug use. She did not have any significant sick contacts or recent travel history.

**Figure 1:**
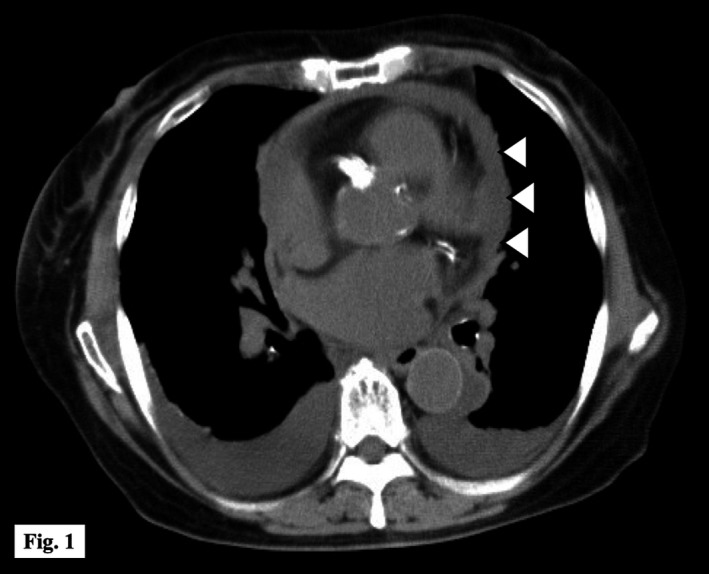
Noncontrast chest CT on admission demonstrated a small volume of pericardial effusion (white arrowhead)

**Figure 2:**
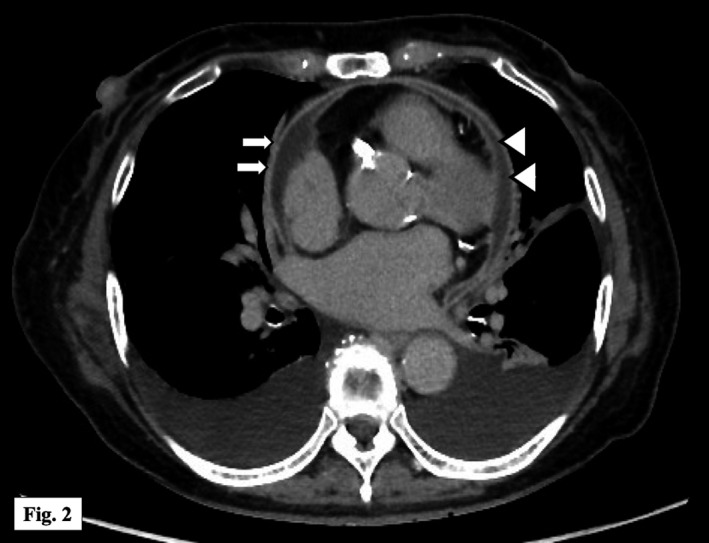
Contrast chest CT on day 10 demonstrated an enlargement of the pericardial effusion (white arrowhead) and thickened pericardium (white arrow)

On physical examination, her temperature was 38.5°C, blood pressure was 120/84 mm Hg, and heart rate was 110 beats per minute. There were jugular venous distention and a slight pericardial friction rub. There was no pulsus paradoxus or pedal edema. Electrocardiogram showed diffuse ST‐segment elevation with subtle PR‐segment depression. Initial white blood cell count was elevated at 19 100/mm^3^, and C‐reactive protein was significantly elevated at 30.5 mg/dL (reference range: <0.3). Cardiac troponin I was also increased at 205.1 pg/mL (reference range: <26.0), while other cardiac biomarkers were within normal limits. Noncontrast chest computed tomography (CT) showed a small pericardial effusion (Figure 1) but transthoracic echocardiography did not show any dynamic signs of cardiac tamponade. A working diagnosis of acute viral pericarditis was made and the patient was admitted to the general medical service. Eighteen hours after admission, she developed septic shock and two sets of blood cultures grew group B streptococcus (GBS). Typically, bacterial pericarditis is seen in immunocompromised patients or in the setting of concurrent pneumonia or maxillofacial infection. This patient had none of these features but as there was no other clear source of infection, bacterial pericarditis was suspected. Ampicillin and clindamycin were prescribed concurrently to reduce risk of toxic shock syndrome. Initially, we were unable to perform diagnostic pericardiocentesis due to technical difficulties. Several days later, serial chest CT showed enlargement of the pericardial effusion with thickened pericardium (Figure 2). A second attempt at pericardiocentesis was successful, and 60 mL of serosanguinous fluid was aspirated, comprising 4050/mm^3^ leukocytes, predominantly lymphocytes (62%). The glucose was 67 mg/dL and lactate dehydrogenase 4088 U/L. Bacterial culture of the pericardial effusion was negative, probably due to prior use of antibiotics. A sample of pericardial fluid was sent to the microbiology laboratory of Tokyo Medical University where purified chromosomal DNA was used for PCR amplification of the 16S rRNA gene and this sequencing was identified as GBS (with 99% accuracy).

The patient in this case did not have signs or symptoms of rheumatologic disease, and serum autoimmune antibodies were negative. There was no preceding viral infection, and serological antibodies for viral pericarditis were negative. Viral PCR analysis was not performed.

Subxiphoid partial pericardiostomy was performed to reduce risk of tamponade. She completed a 4‐week course of intravenous ampicillin. There were no surgical or other complications. A comprehensive screen for underlying malignancy was negative, and we were unable to determine the primary source of infection. The patient made a complete recovery and was discharged. There have been no recurrent symptoms or signs of either pericarditis or cardiac failure.

## 2 DISCUSSION

### 2.1 Current evidence for the use of PCR to diagnose bacterial pericarditis

Antibiotic use prior to culture can conceal the causative microorganism. Recently, PCR has been utilized to identify bacterial pathogens but clinical evidence for the use of PCR in pericardial infections has been limited to sporadic case reports. We performed a literature search to clarify the current use of PCR in the diagnosis of pericarditis. We searched the MEDLINE database with the following keywords: purulent pericarditis, bacterial pericarditis, 16S rDNA sequence, polymerase chain reaction, and PCR. We reviewed all English language articles and excluded case reports where the diagnosis had already been made with conventional tissue or blood culture before PCR. A total of 19 articles (22 cases) dating from 1991 to 2017 were deemed appropriate for further evaluation and included in the summary^1^, ^2^, ^3^, ^4^, ^5^, ^6^, ^7^, ^8^, ^9^, ^10^, ^11^, ^12^, ^13^, ^14^, ^15^, ^16^, ^17^, ^18^, ^19^ (Tables 1 and 2).

**Table 1:** Published cases with culture‐negative bacterial pericarditis caused by gram‐positive and gram‐negative species

Microorganisms	Reference no.	Published year	Age (yo), Gender	Significant backgrounds	Associated conditions	Adjunctive therapies except antibiotics	Complications/Outcome
Gram‐positive species							
*Actinomyces neuii*	1	2006	39, F		Chronic pericarditis	NR	NR/NR
*Nocardia nova*	2	2016	50, F	Alpha‐1‐antitrypsin deficiency and recent lung transplantation	Empyema necessitatis	NA	NA/Survived
*S pneumoniae*	3	2010	22, M		NA	Pericardiostomy after recurrence	Recurrent pericarditis/Survived
	3	2010	45, F		CAP	Pericardial drainage	NA/Survived
	4	2010	57, F		NA	Surgical drainage: NR	Cardiac tamponade/Survived
*Tropheryma whipplei*	5	2013	68, M		Constrictive pericarditis and polyarthritis	Pericardial biopsy and pleural decortication	Fibrosing pleuritis/Sudden death
Gram‐negative species							
*Bartonella henselae*	6	2007	36, M	IV drug abuse and animal contacts	HIV, angiomatous papules, and cryptogenic hepatitis	NR	NR
*Bartonella quintana*	7	2003	41, M	Homeless and alcoholism	Aortic insufficiency	Pericardial biopsy and drainage	NR
*Bordetella holmesii*	8	2011	71, M	DLBCL on Rituximab	NR	Pericardial drainage	NA/Survived
*Campylobacter fetus*	9	2017	62, M		ESRD on hemodialysis	Pericardiocentesis	NA/Survived
*Kingella kingae*	10	2007	43, F		Cardiac tamponade	Pericardial biopsy and drainage	Constrictive pericarditis/Survived
*Helicobacter cinaedi*	11	2007	48, M		Myopericarditis	Pericardial drainage	Inpatient cardiac arrest/Survived

**Table 2:** Published cases with culture‐negative bacterial pericarditis caused by *Mycobacterium* species and others

Microorganisms	Reference no.	Published year	Age (yo), Gender	Significant backgrounds	Associated conditions	Adjunctive therapies except antibiotics	Complications/Outcome
*Mycobacterium* species							
*M tuberculosis*	12	1991	52, F		NR	Pericardiocentesis	NA/Survived
	13	1993	74, F		Advanced atrioventricular block	NR	NA/Survived
	14	1999	23, M	Travels to endemic countries	NR	Pericardiocentesis and pericardiectomy	Recurrent pericarditis/Survived
	15	2001	63, F		Chronic pericarditis	Pericardiocentesis	NA/Survived
	1	2006	42, M		Pneumonia	NR	NR
	1		87, M		Pneumonia	NR	NR
	16	2010	24, F	Pregnant woman	Constrictive pericarditis	Pericardiocentesis	NA/Survived
	17	2015	40, F	Pregnant woman	Cardiac tamponade	Pericardiocentesis	NA/Survived
Others							
*Coccidioides posadasii*	18	2008	35, M		Myopericarditis and heart failure	Pericardial biopsy and surgical drainage	NA/Survived
*Mycoplasma pneumoniae*	19	2002	17, F	Recent bone marrow transplantation	CML and multiple sepsis	Pericardiocentesis	NA/Survived

AIDS, acquired immune deficiency syndrome; ALL, acute lymphocytic leukemia; CAP, community‐acquired pneumonia; CML, chronic myeloid leukemia; DLBCL, diffuse large B‐cell lymphoma; ESRD, end‐stage renal disease; F, female; IVDA: intravenous drug abuse; M, male; NA, not applicable; NR, not reported in detail.

Most patients have already received empiric antibiotics prior to sampling of blood or pericardial fluid, and hence, bacterial cultures are often negative. Atypical organisms are also technically difficult to isolate with conventional microbiology testing which can further delay appropriate treatment. On review of the published cases seen in Tables 1 and 2, both immunocompromised and immunocompetent patients developed atypical bacterial infections.^1^, ^2^, ^6^, ^7^, ^8^, ^10^, ^11^, ^18^ Current laboratory facilities have difficulty identifying *Streptococcus pneumonia*, one of the most common organisms in bacterial pericarditis. Although *S* *pneumoniae* grows rapidly in most conventional automated blood culture systems, it produces autolysin: a cell wall enzyme which causes autolysis during the stationary growth phase. This can distort the appearance of pneumococci on Gram stain and prevent growth on subculture.^3^, ^4^, ^20^

In patients with chronic unexplained pericarditis, PCR identified *Actinomyces neuii*, *Tropheryma whipplei*, and *Mycobacterium tuberculosis*.^1^, ^5^, ^15^, ^16^ Interestingly, most cases of tuberculous pericarditis did not present with typical pulmonary symptoms or miliary tuberculosis. Therefore, diagnosis was delayed and the risk of heart failure and other complications were increased.

Thus, we believe PCR can be beneficial in both identifying the causative microorganism after initiation of empirical antibiotics and detecting uncommon organisms.

However, the utilization of PCR still has limitations such as procedural contamination, accessibility, and cost‐effectiveness.^21^ Due to partial degradation of the DNA, fresh clinical specimens are more accurate than formalin‐fixed tissue.^21^ It is important to remember that the presence of DNA does not necessarily mean persistent infection by the detected microorganism.^22^ Also, PCR cannot determine antimicrobial susceptibility and there are reports of recurrent infection and development of constrictive pericarditis despite completing empirical antibiotic treatment.^9^, ^10^ To our knowledge, this is the first literature review of a PCR strategy for diagnosis of culture‐negative pericarditis and reported cases of PCR‐diagnosed GBS pericarditis.

### 2.2 Risk factors for GBS bacteremia

Jackson et al^23^ reported several chronic conditions which are independently associated with invasive GBS infection: age, cirrhosis, diabetes mellitus, history of cerebrovascular disease, decubitus ulcer, or neurogenic bladder. Most patients seen with GBS bacteremia have at least one of these conditions. In this case, the patient had diabetes mellitus but the primary source of bacteremia was not discovered. In such patients, GBS infection can be considered an “opportunistic infection.”

### 2.3 Therapeutic management of bacterial pericarditis

Current evidence for the use of anti‐inflammatory medications such as aspirin, corticosteroids, and colchicine is limited to viral or immune‐mediated pericarditis. For bacterial pericarditis, most guidelines recommend targeted therapy with antibiotics and pericardial drainage.^24^, ^25^ In this case, anti‐inflammatory medication was not used.

There are several interventional procedures for diagnosis and treatment of pericarditis, including pericardiocentesis, partial, or total pericardiectomy, and pericardiostomy. The ideal choice of procedure depends on the clinical situation. For example, pericardiocentesis is not indicated for all patients but only if there is cardiac tamponade, a large symptomatic pericardial effusion unresponsive to medical therapy, or for evaluation of suspected bacterial or neoplastic etiology.^25^ Fluoroscopic or echocardiographic guidance decreases the risk of complications such as coronary artery or cardiac cavity puncture, hemothorax, or hepatic injury.^24^ Clinicians must consider the risk‐benefit for each patient. Pericardiocentesis can also help with diagnosis, but biopsy specimens may be insufficient. Also, although it may provide temporary symptomatic relief, more extensive procedures such as pericardiectomy or pericardiostomy are sometimes required.^24^ In the modern antibiotic era, the development of constrictive pericarditis requiring pericardiectomy is uncommon. In cases which have progressed to constrictive pericarditis, there may be heavy calcification and involvement of the visceral pericardium, which complicates surgical procedures, and the perioperative mortality of pericardiectomy remains high at 4%‐10%.^26^, ^27^, ^28^ Therefore, pericardiectomy is avoided unless absolutely necessary.

Regarding subxiphoid pericardiostomy, Becit et al published a large case series of 368 patients with bacterial pericarditis documenting the safety and effectiveness of this procedure; perioperative mortality was 0% and overall 30‐day mortality was 0.8%. Becit et al^29^ also highlighted the importance of a multidisciplinary team approach including cardiothoracic surgeons to aid in making appropriate and timely management decisions.

In summary, PCR can identify both typical and atypical microorganisms and, with careful interpretation, represents a promising new diagnostic test for culture‐negative pericarditis. Physicians managing patients with pericarditis should consult with cardiology and cardiothoracic surgery teams to help decide the most timely and appropriate interventions.

## CONFLICT OF INTEREST

All authors declare that they have no conflict of interest.

## AUTHOR CONTRIBUTION

All authors participated in drafting the article and revising it critically for intellectual content. TT, NI, KA, YM, SS, and KH: interpreted data. TT, NI, YM, and SS: provided medical care. KH: performed diagnostic pericardiocentesis. AY: performed elective pericardiostomy. KO: performed quantitative polymerase chain reaction. SM and YS: supervised this case. IC: provided extensive editing of this paper and advice on the literature summary.

## VERIFICATION

All authors have access to the data and have contributed significantly to writing this manuscript.
